# Three years experience with dried blood spot α-glucosidase screening for Pompe disease in British Columbia, Canada

**DOI:** 10.1186/1471-2474-14-S2-P2

**Published:** 2013-05-29

**Authors:** Gabriella Horvath, Sandra Sirrs, Sylvia Stockler, Ramona Salvarinova-Zivkovic, Hilary Vallance, Paula Waters

**Affiliations:** 1 British Columbia Children's Hospital, Vancouver, British Columbia, Canada

## Introduction

Pompe disease (OMIM #232300) or glycogen storage disease type II is an autosomal recessive lysosomal storage disease caused by mutations in the glucosidase alpha acid (GAA) gene. The acid alpha-glucosidase enzyme is required for the degradation of cellular glycogen, and its reduced activity results in accumulation of glycogen in muscle and cardiac tissues with variable clinical presentation. Demonstration of deficient acid alpha-glucosidase (GAA) enzyme activity is diagnostic, and molecular testing is available for confirmation or clarification. As Pompe disease is in the differential diagnosis of a wide variety of myopathies, simple first-line tests are needed. Use of dried blood spots (DBS) has logistical advantages over the traditional approach of enzyme assay in isolated lymphocytes, and enzyme stability permits DBS shipment to a central laboratory.

## Methods/results

Patients were referred for enzyme testing who presented with muscle weakness, muscle pain, respiratory insufficiency, and/or cardiomyopathy in infancy. Dried blood spot (DBS) acid α-glucosidase testing, with neutral α-glucosidase as a control enzyme, was measured using a previously described fluorimetric method [1,2]. Out of 149 samples tested, three cases of Pompe disease were detected by DBS assay during a three year period. Two patients with low values for acid α-glucosidase in DBS were confirmed to carry hypomorphic alleles not associated with clinical disease. Two patients with low values, overlapping those of the two patients with hypomorphic alleles, had no mutations detected. Two further patients had normal results on a second DBS card, suggesting that the initial blood spots might have been compromised.

## Conclusion

Since the introduction of the DBS alpha-glucosidase method, several new Pompe cases have been diagnosed at our centre. A repeat DBS should be requested to confirm initial low results before proceeding to further testing. In a significant proportion of false positive cases, benign hypomorphic alleles provide an explanation for reduced activity of acid α-glucosidase.
